# Angiopoietin-2 expression and its relationship with lymphangiogenesis and clinicopathological characteristics in cutaneous malignant melanoma

**DOI:** 10.3389/fonc.2023.1113604

**Published:** 2023-07-13

**Authors:** Wei Zheng, Wei Ju, Xi-Hu Yang, Zhi-Xin Yan

**Affiliations:** ^1^ Department of Burns and Plastic Surgery, Affiliated Hospital of Jiangsu University, Zhenjiang, Jiangsu, China; ^2^ Department of Oral and Maxillofacial Surgery, Affiliated Hospital of Jiangsu University, Zhenjiang, Jiangsu, China

**Keywords:** cutaneous malignant melanoma (CMM), angiopoietin 2 (Ang-2/ANGPT2), lymphatic vessel density (LVD), lymphatic vessel endothelial hyaluronan receptor-1 (LYVE-1), lymphatic metastasis

## Abstract

**Objective:**

The aim of this study was to investigate angiopoietin-2 (Ang-2/ANGPT2) expression and its relationship with lymphangiogenesis and clinicopathological characteristics in cutaneous malignant melanoma (CMM).

**Methods:**

Gene expression differences between metastatic melanoma and melanoma *in situ* in 472 patients from the TCGA database were analyzed. The target gene Ang-2 was screened. A clinical study was conducted to analyze the correlation between Ang-2 expression in CMM and tumor-associated lymphangiogenesis. A total of 42 patients with primary CMM who underwent extended tumor resection procedures at the Affiliated Hospital of Jiangsu University were included in this study. Clinical data (gender, age, lymph node metastasis, Breslow thickness, and clinical stage) were collected. The expression levels of both Ang-2 and lymphatic vessel endothelial hyaluronan receptor-1 (LYVE-1) proteins were detected by immunohistochemistry (IHC). Lymphatic vascular density (LVD) was counted by using LYVE-1 to label lymphatic endothelial cells (LECs) in peritumoral and intratumoral areas per high-magnification field of view. Statistical analysis was performed using the Pearson correlation test and Student’s *t*-test.

**Results:**

Using the TCGA database, it was found that the gene expression level of Ang-2 in 368 cases of metastatic melanoma was significantly higher than that in 104 cases of melanoma *in situ*. Correlation analysis showed a significant relationship between Ang-2 and LYVE-1, and vascular endothelial growth factor receptor 3(VEGFR3) expression, respectively, in CMM. Moreover, the optimal cutoff value of survival analysis showed that high Ang-2 expression in CMM had a worse prognosis, based on data from the TCGA database. Our research showed that Ang-2 was more highly expressed in the group of patients with lymph node metastasis and in the group of stage 3C-4 patients than in the group of patients with no lymph node metastasis and in the group of stage 0-3B patients. Our research also showed that LVD in the group of patients with lymph node metastasis and in the group of stage 3C-4 patients was significantly higher than that in the group of no lymph node metastasis and in the group of stage 0-3B patients, respectively. Breslow thickness also correlated with Ang-2 expression and LVD. Ang-2 expression was not related to sex or age. Ang-2 expression was obviously correlated with LVD.

**Conclusion:**

An evaluation of Ang-2 expression and LVD can be used to predict the risk of tumor lymphatic metastasis and determine the prognosis of CMM. These results may also provide a new clinical treatment strategy for CMM.

## Introduction

In recent decades, the incidence and mortality rates of cutaneous malignant melanoma (CMM) have been increasing on a global scale. According to the literature, the global incidence of melanoma was approximately 3.9 per 100,000 in 2017, representing a 41.2% increase since 1990 (1, 2). CMM is one of the most aggressive neoplasms, with a tendency to metastasize early in regional lymph nodes. The spread of CMM via lymphatic vessels is considered the preferred route of metastasis (3, 4). The crosstalk between melanoma cells and their microenvironment can promote tumor lymphangiogenesis. It has been confirmed that melanoma cells can release a series of cytokines, including VEGF-C, nerve growth factor receptor (NGFR), CXCL5, CD147, and Apelin, for lymphatic remodeling and lymphangiogenesis (5–10). Previous studies have proven that angiopoietin 2 (Ang-2/ANGPT2) can regulate lymphatic vessel development, and mutations in the ANGPT2 gene were recently found in human primary lymphedema (11–13); however, it has not been studied in CMM. Therefore, the relationship between Ang-2 and tumor lymphatic metastasis of CMM and lymphangiogenesis is still unclear. This study aimed to evaluate Ang-2 expression and lymphatic vascular density (LVD) in tumoral areas of CMM, assess the relationship between Ang-2 expression with lymphangiogenesis and clinicopathological characteristics, and judge the correlation between LVD and clinicopathological characteristics in 42 cases of CMM.

## Materials and methods

### Bioinformatics analysis

Studies with the presence of melanoma *in situ* as the control analyzed the gene expression difference between melanoma *in situ* and metastatic melanoma by the Cancer Genome Atlas (TCGA) database. Subsequently, we combined the differentially expressed genes to obtain the target gene signatures. To determine whether the ANGPT2/Ang-2 gene was related to prognostic value, survival analysis was performed in the R environment.

### Patients and tumor specimens

In this study, 42 patients with primary CMM who underwent tumor extended resection procedures at the Affiliated Hospital of Jiangsu University from October 2013 to January 2022 were screened. All CMM specimens were paraffin-embedded and sliced. A routine histological examination was performed with hematoxylin–eosin staining. The patients’ ages ranged from 42 to 90 years, and none of them had received preoperative radiation therapy or chemotherapy. The TNM staging of melanoma is classified according to the World Health Organization (WHO) criteria (14, 15). The clinical characteristics of the patients are summarized in **Table 1**. Two cases of normal skin tissue were selected as controls. The ethics committee of the Affiliated Hospital of Jiangsu University approved the study.

**Table 1:** Relationship between Ang-2 expression and clinical characteristics in 42 cases of CMM.

Variable	*N*	High expression (cases, %)	*χ* ^2^	*p*
Age (years)				
<65	15	8 (53.33)	0.371	0.744
≥65	27	17 (62.96)		
Gender				
Male	25	16 (64.00)	0.541	0.534
Female	17	9 (52.94)		
LN metastasis				
Yes	22	18 (81.82)	7.687	0.006
No	20	7 (35.00)		
Clinical stage				
Stage 0-3B	23	8 (34.78)	10.747	0.001
Stage 3C-4	19	17 (89.47)		
Breslow thickness(mm)				
≥2	18	16 (88.88)	9.242	0.002
<2	24	9 (37.50)		

N, number of specimens; LN, lymph node.

### Immunohistochemistry

Immunohistochemistry (IHC) staining was performed using the streptavidin-peroxidase conjugate method. Briefly, 4-mm paraffin sections were deparaffinized, rehydrated, and then incubated with fresh 3% hydrogen peroxide for 15 min at room temperature. After rinsing with PBS, the tissue sections were subjected to heat-induced epitope retrieval. Goat serum (10%) was used to block nonspecific binding for 15 min at room temperature. The cells were incubated with Ang-2 monoclonal antibody (1:2,000, Abcam, UK) and LYVE-1 monoclonal antibody (1:2,000, Abcam, UK) overnight at 4°C. The next day, the cells were incubated with HRP goat anti-rabbit IgG (Proteintech Biotechnology Inc., China) for 30 min at room temperature. After rinsing with PBS, the sections were stained with fresh 3,3′-diaminobenzidine (DAB; Proteintech Biotechnology Inc., China), counterstained in hematoxylin, dehydrated, and mounted.

### Evaluation of IHC

Using light microscopy, two pathologists independently evaluated the IHC stains of the patients as previously described (16, 17), with no knowledge of clinical data. Ang-2 was stained in the cytoplasm or membrane, mainly in the cytoplasm in CMM. The whole film was observed at low magnification to determine the infiltration edge of the tumor. Then, five high-magnification (400×) fields were selected to determine the percentage of stained cells for comprehensive scoring. The percentage of positive cells was graded as described previously (18) in CMM. In brief, 0%–10%, 11%–50%, and higher than 50% of positive cells were considered to be negative (−), medium (+), and positive (++), respectively. Both negative expression and medium expression were regarded as low expression, and positive expression was regarded as high expression.

The five highest number of lymphatic vessel fields of view were chosen to count lymphatic vessels by using LYVE-1 antibody, and the average lymphatic vascular number/high-magnification field of view was defined as LVD.

### Statistical analysis

Each experiment was performed independently twice. All statistical analyses were performed using SPSS 26.0 software. Data are expressed as the means ± SDs. The relationship between Ang-2 expression and clinical characteristics was determined using the chi-squared test. The relationship between LVD and clinical characteristics was determined by two independent samples *t*-tests. The correlation between Ang-2 expression and LVD was analyzed by Pearson correlation analysis. A *p*-value < 0.05 was considered significant.

## Results

Using bioinformatics analysis, we suspected that Ang-2 might act as a key cytokine that participates in lymphangiogenesis, which is consistent with our previous study (19). To further explore our hypothesis, a series of clinicopathological experiments were performed. Our results showed that the degree of Ang-2 expression correlated with clinical stage, lymph node metastasis, and LVD. Our findings enrich the mechanism of lymphangiogenesis in CMM and may provide new molecular markers for CMM therapy.

### Ang-2 gene expression profile was increased in metastatic CMM

**Figure 1:**
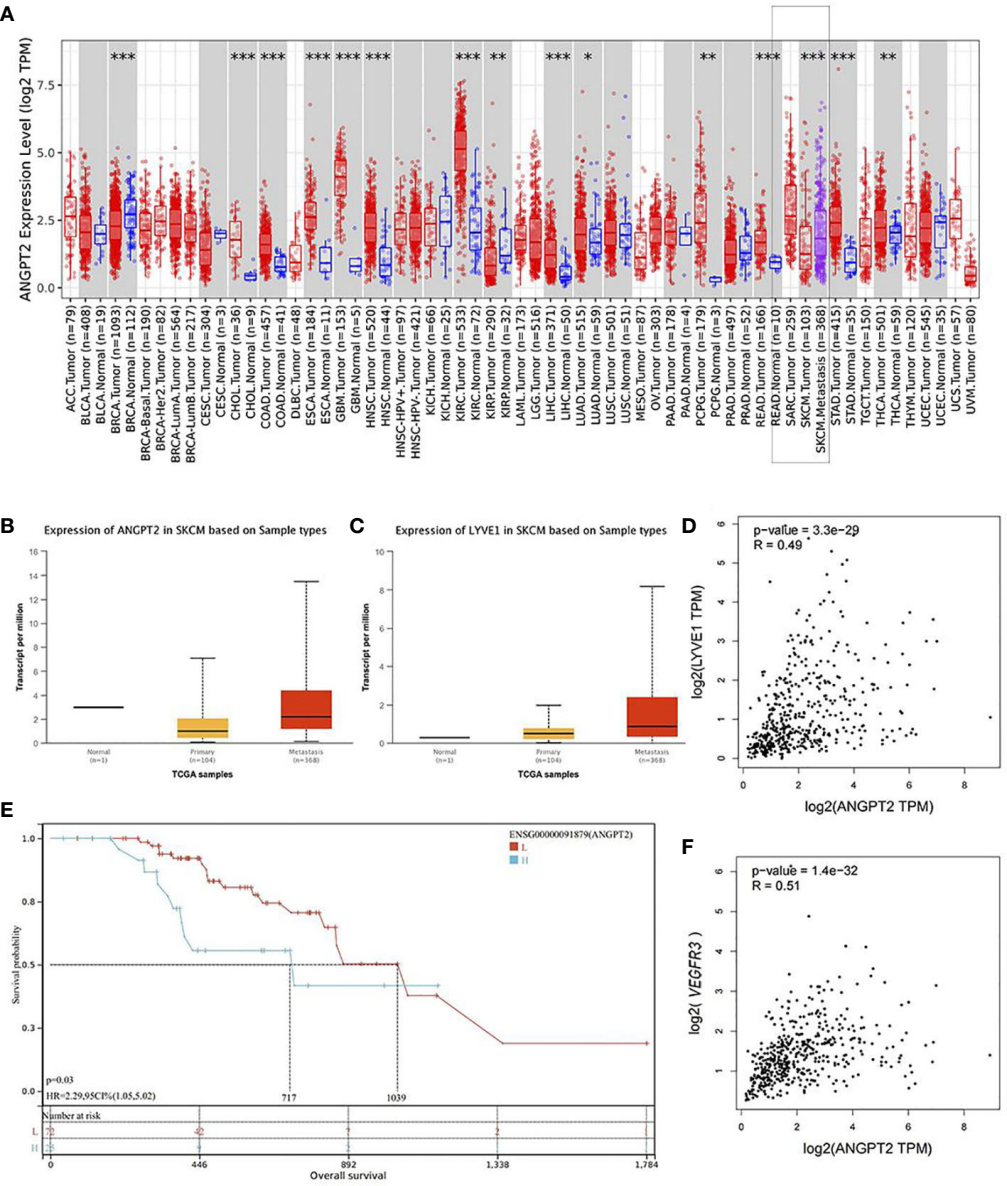
Ang-2/ANGPT2 gene expression of melanoma in the database. ANGPT2 expression in metastatic melanoma (*n* = 368) was found to be significantly higher than that in melanoma *in situ* (*n* = 103) by searching the TCGA database, skin cutaneous melanoma (SKCM), *p* < 0.001 **(A, B)**. LYVE-1 was more highly expressed in metastatic melanoma (*n* = 368) **(C)**. Correlation analysis showed that there was a significant correlation between Ang-2 and LYVE-1/VEGFR3 in melanoma, *p* < 0.01 **(D, F)**. Kaplan–Meier survival curves were generated for the comparison of groups of high (blue line) and low (red line) Ang-2 expression, *p* = 0.03 in the log-rank test **(E)** (*p value < 0.05; **p value <0.01; ***p value <0.001).

The Ang-2 gene expression profile in 368 cases of metastatic melanoma was found to be significantly higher than that in 103 cases of melanoma *in situ* by searching the TCGA database (**Figures 1A, B**). The LYVE-1 gene was also more highly expressed in metastatic melanoma (**Figure 1C**). Correlation analysis showed that there was a significant correlation between Ang-2 and LYVE-1/VEGFR3 expression in CMM, *p* < 0.01 (**Figures 1D, F**). Therefore, we speculate that there is a relationship between Ang-2 expression and LVD/lymphangiogenesis in CMM. The optimal cutoff value of survival analysis showed that high Ang-2 expression in CMM was associated with a worse prognosis (*p* = 0.03) (**Figure 1E**).

### Relationship between Ang-2 and clinical features of CMM

**Figure 2:**
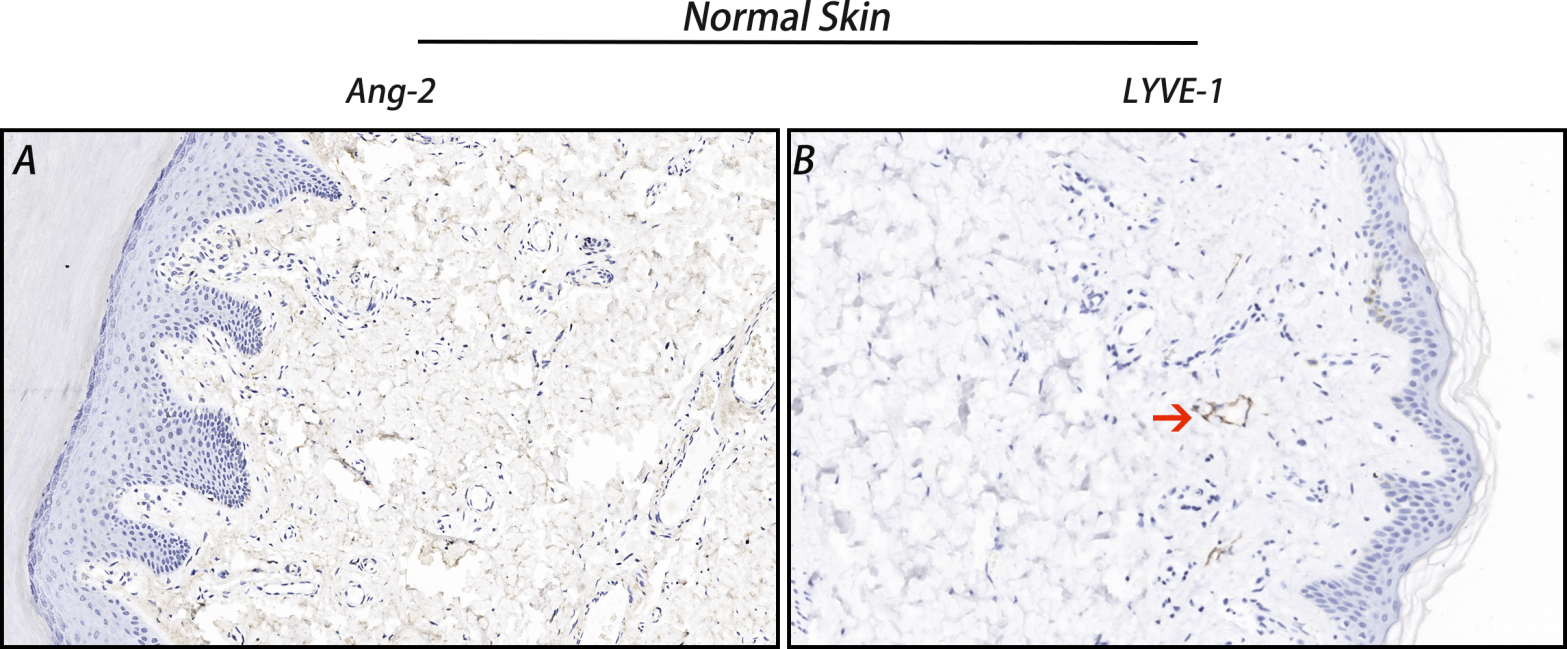
Ang-2 and LYVE-1 expression in normal skin. Ang-2 was negatively expressed in normal skin **(A)**, and LYVE-1-stained lymphatic vessels were observed in normal skin **(B)**. Red arrow: lymphatic vessel.

**Figure 3:**
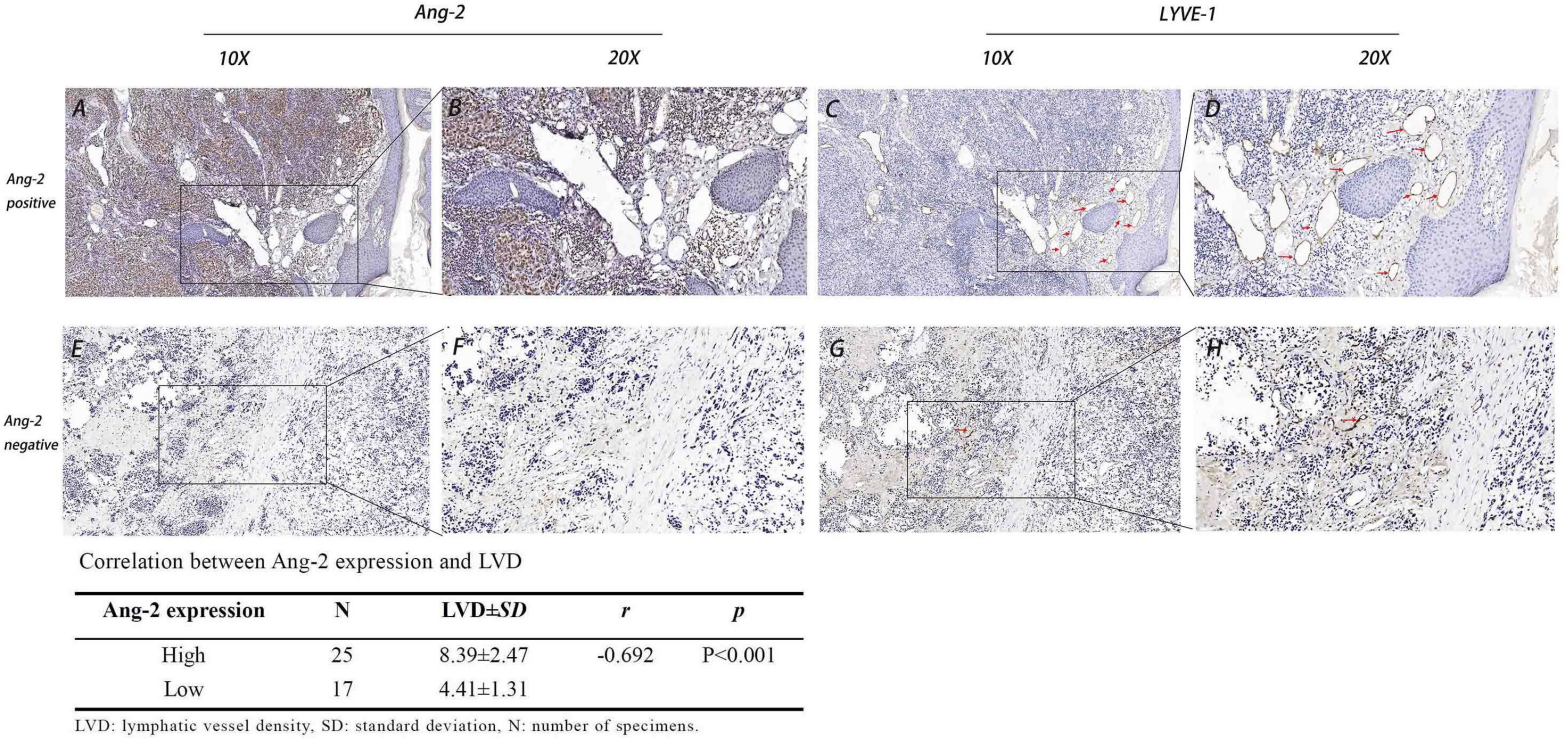
Expression of Ang-2 and LYVE-1 and the correlation between Ang-2 expression and LVD in CMM. High Ang-2 expression in tumor tissue (**A**: 10×, **B**: 20×). LYVE-1 labeled lymphatic vessels in Ang-2 high expression tissue (**C**: 10×, **D**: 20×). Low Ang-2 expression in tumor tissue (**E**: 10×, **F**: 20×). LYVE-1 labeled lymphatic vessels in Ang-2 low expression tissue (**G**: 10×, **H**: 20×). Red arrows: lymphatic vessel.

The IHC staining results showed that Ang-2 expression was negative in normal skin tissues (**Figure 2**), but in most of the 42 cases of CMM, Ang-2 was strongly positively stained in the cytoplasm and membrane, mainly in the cytoplasm (**Figures 3E, F**). The high expression rate of Ang-2 was 59.52% in 42 patients with CMM (**Table 1**). The high expression rate was 81.82% in the lymphatic node metastasis group, which was significantly higher than that in the nonlymphatic node metastasis group (35.00%) (*p* = 0.006) (**Table 1**). The expression rate of Ang-2 in the stage 3C-4 group was 89.47%, which was significantly higher than that in the stage 0-3B group (34.78%) (*p* < 0.001) (**Table 1**). High Ang-2 expression was also correlated with Breslow thickness (*p* = 0.001). In contrast, Ang-2 expression showed no significant correlation with age or sex (*p* > 0.05) (**Table 1**).

### Relationship between LVD and clinical features of CMM

Using the lymphatic endothelial cell-specific marker LYVE-1, lymphatic vessels in the CMM tissue slice were detected. LYVE-1-positive lymphatic vessels were surrounded by inflammatory cells in the peritumoral stroma (**Figures 3C, D**). LVD in the lymph node metastasis group (8.15 ± 2.94) and in the stage 3C-4 group (9.38 ± 2.07) was significantly higher than that in the no lymph node metastasis group (5.27 ± 1.85) and in the stage 0-3B group (4.82 ± 1.43), respectively, all *p* < 0.05 (**Table 2**). The LVD in the Breslow thickness ≥ 2 mm group (9.30 ± 2.02) was significantly higher than that in the Breslow thickness < 2 mm group (4.69 ± 1.34), *p* < 0.001 (**Table 2**).

**Table 2:** Relationship between LVD and clinical characteristics in 42 cases of CMM.

Variable	*N*	LVD *±* SD	*F*	*t*	*p*
Age (years)					
<65	15	6.72 ± 3.00	0.009	−0.091	0.928
≥65	27	6.81 ± 2.82			
Gender					
Male	25	6.99 ± 2.85	0.013	0.576	0.568
Female	17	6.47 ± 2.91			
LN metastasis					
Yes	22	8.15 ± 2.94	–	3.834	<0.001
No	20	5.27 ± 1.85			
Clinical stage					
Stage 0-3B	23	4.69 ± 1.34	6.016	−8.827	<0.001
Stage 3C-4	19	9.30 ± 2.02			
Breslow thickness(mm)					
≥2	18	9.38 ± 2.07	4.669	8.439	<0.001
<2	24	4.82 ± 1.43			

LVD, lymphatic vessel density; SD, standard deviation; N, number of specimens; LN, lymph node.

### Relationship between Ang-2 expression and LVD in CMM

In all cases, the LVD in the Ang-2 high-expression group (8.39 ± 2.47) was significantly higher than that in the Ang-2 low-expression group (4.41 ± 1.31). Pearson correlation analysis showed that Ang-2 expression was positively correlated with LVD, *r* = −0.692, *p* < 0.01 (**Figure 3**).

## Discussion

CMM tends to metastasize, spread, and access regional lymph nodes preferentially through lymphatic vessels, even at the early stage (4, 20). Hence, lymphangiogenesis and lymph node metastasis are important determinants in the prognosis and clinical management of melanomas. The typical therapy for malignant melanoma is surgical excision, immunotherapy, targeted gene therapy, and chemotherapy, but the mortality of this disease has not significantly improved (21). Therefore, a more effective treatment strategy or a more sensitive prognostic indicator is urgently needed.

VEGF-C and VEGF-D are well-known lymphangiogenesis stimulators, and they were shown to promote melanoma lymph node metastasis through the activation of VEGFR-3 on lymphatic vessel endothelial cells (12, 22, 23). As lymphatic spread is a complex, multistep process, several different biomarkers must be combined to define new prognostic subgroups in cutaneous melanoma (24, 25). In addition to the vascular endothelial growth factor (VEGF) receptor pathway, angiopoietins are a family of growth factors that play a key role in cardiovascular and lymphatic development, both under physiologic and pathologic conditions (13, 19, 26). Ang-2 regulates the formation of tumor lymphatic vessels, promoting tumor proliferation, infiltration, and metastasis (11–13, 27); thus, Ang-2/Tie was considered another specific marker for stimulating lymphangiogenesis (26, 28). In addition to pro-angiogenesis or lymphangiogenesis, melanoma cell-derived Ang-2 was much higher in metastatic specimens than in either nevus or primary melanomas in an array of clinical and preclinical studies (29). However, the specific role of Ang2 in melanoma lymphatic processes remains unclear. To verify the relationship between Ang-2 and lymphangiogenesis/tumor metastasis in CMM, we conducted relevant research.

In the first step of our study, we used the TCGA database to determine the gene expression level of Ang-2 in metastatic melanoma and *in situ* melanoma. The results showed that the Ang-2 gene had significantly higher expression in metastatic melanoma, and patients who expressed a higher level of Ang-2 had an adverse prognosis. Ang-2 gene expression correlated with LYVE-1 expression in CMM, which indicated that Ang-2 might promote lymphangiogenesis and tumor progression in melanoma.

Next, we performed IHC staining on CMM samples, which showed that Ang-2 was highly expressed in the stage 3C-4 group and the lymph node metastasis group, suggesting that Ang-2 may be involved in the infiltration and metastasis of CMM. LVD in the high Ang-2 expression group was significantly higher than that in the low Ang-2 expression group, suggesting that Ang-2 might promote tumor lymphangiogenesis and be closely related to tumor lymph node metastasis. Past studies have found that Ang-2 was associated with lymphangiogenesis in cardiovascular disease and that higher Ang-2 expression was positively associated with lymph node metastasis in breast cancer patients (12, 30, 31). To our knowledge, this is the first report that Ang-2 expression is associated with lymphangiogenesis and tumor lymph node metastasis in progressive CMM. Therefore, targeted blockade of Ang-2 expression may show promise for treating CMM and interrupting lymphatic metastasis. LYVE-1 is expressed on the lymphatic vessel endothelium and is considered to be a specific marker of lymphatic vessels (32). In this study, LVD in the lymph node metastasis group and the stage 3C-4 group was higher than that in the no lymph node metastasis group and stage 0-3B group, respectively. We also further verified that LVD is closely related to the development of CMM and lymph node metastasis. The LVD in the Ang-2 high-expression group was significantly greater than that in the Ang-2 low-expression group, also suggesting that Ang-2 plays an important role in tumor lymphangiogenesis. However, the mechanism by which Ang-2 regulates lymphangiogenesis in melanoma needs further in-depth study.

Collectively, our data demonstrate that Ang-2 expression is closely correlated with clinical stage and lymph node metastasis in CMM, which contains several different clinical subtypes, with acral lentiginous melanoma being the most common type in Asia. Patients with this subtype of CMM have a worse prognosis than those with the common subtypes in Europe or the United States (33). Out of 42 cases, there were only two cases in our study that were definitively nonacral origin melanoma, three other cases were first diagnosed as melanoma from the inguinal LN, and all remaining cases were acral lentiginous melanomas. According to our research, Ang-2 was strongly positively expressed in acral melanoma tissue. This may be related to the late staging or adverse prognosis of the patients. Based on the relationship between Ang-2 and LVD in CMM, we speculate that Ang-2 may promote tumor-associated lymphangiogenesis, and these results may indicate the feasibility of a new prognostic indicator and a new strategy for the clinical treatment of CMM.

## Data availability statement

The datasets presented in this study can be found in online repositories. The names of the repository/repositories and accession number(s) can be found in the article/supplementary material.

## Ethics statement

The studies involving human participants were reviewed and approved by Ethics Committee of Affiliated Hospital of Jiangsu University. The patients/participants provided their written informed consent to participate in this study.

## Author contributions

Conceived and designed the study: ZX-Y. Collect and collate case: WZ, WJ, XH-Y, ZX-Y. Analyzed the data: WZ, WJ, XH-Y, ZX-Y. Wrote the manuscript: WZ, WJ, ZX-Y. All authors contributed to manuscript revision, read, and approved the submitted version.
